# ABCC6 deficiency promotes dyslipidemia and atherosclerosis

**DOI:** 10.1038/s41598-021-82966-y

**Published:** 2021-02-16

**Authors:** Christopher Brampton, Viola Pomozi, Li-Hsieh Chen, Ailea Apana, Sara McCurdy, Janna Zoll, William A. Boisvert, Gilles Lambert, Daniel Henrion, Simon Blanchard, Sheree Kuo, Georges Leftheriotis, Ludovic Martin, Olivier Le Saux

**Affiliations:** 1 Department of Cell and Molecular Biology, John A. Burns School of Medicine, University of Hawaii, 651 Ilalo St. BSB222E, Honolulu, HI USA; 2 Bio-Rad Laboratories, Inc., Hercules, CA USA; 3 Department of Medicine, John A. Burns School of Medicine, University of Hawaii, Honolulu, HI USA; 4 Department of Medicine, University of California San Diego, San Diego, USA; 5 University of La Réunion Medical School (France) INSERM UMR1188 DéTROI, Ste Clotilde, La Réunion, France; 6 MITOVASC Institute - UMR CNRS 6015 INSERM U1083, University of Angers, Angers, France; 7 Département d’Immunologie et d’Allergologie, University Hospital of Angers, 49000 Angers, France; 8 Inserm U1232, CRCINA, University of Angers, 44000 Nantes, France; 9 Department of Pediatrics Kapi’olani Medical Center for Women and Children, University of Hawaii, Honolulu, HI USA; 10 Faculty of Medicine, University of Nice-Sophia Antipolis, 06107 Nice, France; 11 Laboratory of Physiology and Molecular Medicine (LP2M) UMR CNRS 7073, 06107 Nice, France; 12 PXE Consultation Center, MAGEC Reference Center for Rare Skin Diseases, Angers University Hospital, Angers, France; 13 BNMI, CNRS 6214/INSERM 1083, University Bretagne-Loire, Angers, France

**Keywords:** Skin diseases, Calcification, Atherosclerosis, Calcification, Peripheral vascular disease, Experimental models of disease

## Abstract

ABCC6 deficiency promotes ectopic calcification; however, circumstantial evidence suggested that ABCC6 may also influence atherosclerosis. The present study addressed the role of ABCC6 in atherosclerosis using *Ldlr*^−/−^ mice and pseudoxanthoma elasticum (PXE) patients. Mice lacking the *Abcc6* and *Ldlr* genes were fed an atherogenic diet for 16 weeks before intimal calcification, aortic plaque formation and lipoprotein profile were evaluated. Cholesterol efflux and the expression of several inflammation, atherosclerosis and cholesterol homeostasis-related genes were also determined in murine liver and bone marrow-derived macrophages. Furthermore, we examined plasma lipoproteins, vascular calcification, carotid intima-media thickness and atherosclerosis in a cohort of PXE patients with ABCC6 mutations and compared results to dysmetabolic subjects with increased cardiovascular risk. We found that ABCC6 deficiency causes changes in lipoproteins, with decreased HDL cholesterol in both mice and humans, and induces atherosclerosis. However, we found that the absence of ABCC6 does not influence overall vascular mineralization induced with atherosclerosis. Decreased cholesterol efflux from macrophage cells and other molecular changes such as increased pro-inflammation seen in both humans and mice are likely contributors for the phenotype. However, it is likely that other cellular and/or molecular mechanisms are involved. Our study showed a novel physiological role for ABCC6, influencing plasma lipoproteins and atherosclerosis in a haploinsufficient manner, with significant penetrance.

## Introduction

Vascular calcification is common in aging and can also result from diabetes, end-stage renal disease, atherosclerosis, or inherited disorders. Clinical evidence demonstrated that cardiovascular calcification is a significant risk factor for cardiovascular events^1^. ABCC6 is a member of the ATP binding cassette superfamily^2^ and is a major regulator of ectopic calcification^3,4^. ABCC6 regulates mineralization by modulating an extracellular purinergic pathway through several key enzymes that generate inorganic pyrophosphate (PPi) and prevent its degradation^5–7^. Indeed, PPi is a potent inhibitor of ectopic calcification^8^. The cellular function of ABCC6 accounts for ~ 60% of plasma PPi levels in humans and mice^9,10^. The highest levels of *ABCC6* expression are found in liver and kidneys, though other cell types also express the protein^11,12^. Inherited ABCC6 deficiency causes dermal, ocular and cardiovascular calcification in pseudoxanthoma elasticum (PXE) patients^4^, some cases of generalized arterial calcification of infancy (GACI)^13^ and is likely responsible for the PXE-like manifestations in β-thalassemia patients^14,15^.

Multiple lines of evidence indicate a pathologic correlation between ABCC6 function and dyslipidemia. Pisciotta et al*.* reported a PXE patient carrying a heterozygous *LDLR* mutation (p.R574H) with severe vascular stenosis and calcification^16^. The dystrophic cardiac calcification phenotype (DCC) associated with *Abcc6* deficiency in mice^17,18^ was first identified in response to high fat diets^19,20^. Quantitative trait loci analysis identified *Abcc6* as one of the root causes of atherosclerosis and aortic calcification induced by hyperlipidemia in *ApoE*^−/−^ mice^21^. Furthermore, previous publications have presented suggestive evidence, but not proven, that PXE patients are susceptible to increased atherosclerosis ^22–24^.

In this study, we explored the possibility that beyond calcification, ABCC6 dysfunction could also play a significant role in dyslipidemia and atherosclerosis. For this purpose, we crossed *Abcc6*^−/−^ mice into an atherogenic *Ldlr*^−/−^ background and compared the results of our investigation with a cohort of human PXE patients. We found that reduced ABCC6 function indeed leads to dyslipidemia and enhanced atherosclerosis in both *Abcc6*-deficient mice and in PXE patients. Vascular calcification was elevated in both *Abcc6*^+*/−*^; *Ldlr*^−/−^ and *Abcc6*^−/−^*; Ldlr*^−/−^ animals, but there was no significant difference with *Ldlr*^−/−^ control mice, suggesting that the *Abcc6* genotype has no effect on atherosclerotic mineralization. Overall, our data imply that changes in HDL levels and/or composition as well as systemic inflammation act as contributors to the atherosclerotic phenotype seen in the *Abcc6*^−/−^ mouse model and PXE patients.

## Material and methods

The data that support the findings of this study are available from the corresponding author upon reasonable request. Because of the sensitive nature of the data from human subjects in this study, these may be subjected to approval by the authors who provided them (LM and GL).

### Animals

*Abcc6*^*tm1Aabb*^ mice were generated on a 129/Ola background^25^ and backcrossed into a C57BL/6J > 10 times. *Ldlr*^*tm1Her*^ mice in the C57BL/6 background were provided by WAB who purchased them from The Jackson Laboratory (Bar Harbor, ME). These mice are hereafter designated as *Abcc6*^−/−^ and *Ldlr*^−/−^, respectively. By simple crosses, we have generated *Abcc6* haploinsufficient (*Abcc6*^+*/−*^;*Ldlr*^−/−^) and double knockout (*Abcc6*^−/−^*;Ldlr*^−/−^) animals to determine if a gene dosage affects atherosclerosis and associated calcification. *Ldlr*^−/−^ mice with normal *Abcc6* expression were used as controls. In this study, both male and female, age-matched mice were used, as gender had no significant impact on results. All mice were housed in pathogen-free rooms in AAALAC-accredited animal facilities at the University of Hawaii School of Medicine. Mice were kept under routine laboratory conditions with 12-h light–dark cycle with ad libitum access to water and either a standard chow diet or an atherogenic high fat diet (HFD) that contained 15.8% (wt/wt) fat, 1.25% (wt/wt) cholesterol and no cholate (diet #94059; Harlan Teklad). The University of Hawaii Institutional Animal Care and Use Committee approved these studies. Experiments were conducted according to the NIH Guide for the Care and Use of Laboratory Animals.

### PXE patients and healthy controls

Fasting plasma samples and phenotypical data for PXE patients were obtained from the MAGEC PXE Reference Center of the Angers University Hospital, France. The diagnosis of PXE was based on a combination of established criteria for indisputable PXE, including clinically suggestive skin lesions, angioid streaks and histologically proven fragmented and calcified elastic fibers on skin biopsy^26^. For lipoprotein analyses, PXE and controls plasma samples came from subjects that were not treated with cholesterol lowering medication. Control plasma samples came from a group matched for gender and age without evidence of PXE or other pathology.

For atherosclerotic plaques assessment, results were compared to the NUMEVOX cohort. This cohort of subjects with at least one metabolic syndrome criterion with or without sleep apnea or liver steatosis is registered on clinicaltrials.gov (NCT00997165). The institution review board of the Angers University Hospital, France has approved these studies and the participants have given informed consent when required. All methods were carried out in accordance with relevant guidelines and regulations.

### Calcification measurements

The level of mineralization in aortic, whisker and heart tissues was quantified using a colorimetric assay^27^ that directly measures the amount of excess calcium, which is normalized to the weight of the excised tissues, as described^18^, and expressed in mg/dL per milligram of tissue. The aorta comprised the aortic arch and the thoracic and abdominal sections, including the iliac bifurcation.

### Atherosclerosis assessment

*A*fter 16 weeks of atherogenic diet, blood was collected from mice under anesthesia through cardiac puncture and the animals were euthanized using standard CO_2_ procedures. The animals were perfused with cold phosphate buffered saline (PBS) and a solution of 4% paraformaldehyde and 5% sucrose. After excising, aortas were cleaned of adventitial fat and opened longitudinally from the iliac bifurcation right to the common carotid artery. Aortas were pinned out onto a black wax board and stained for lipid with Oil Red O. The stained areas and overall inner surface of aortas were measured using Image J software (NIH) and the percent area stained with Oil Red O was determined.

### Plasma lipoproteins and lipid analyses

#### Triglycerides and total cholesterol

These were either measured in fasting human samples by the clinical laboratory services at the Angers University Hospital, France, or in fasting animal samples using quantification kits from Abcam (Ab65336, Cambridge, MA) and from Wako Diagnostics (439-17501, Mountain View, CA). Apolipoprotein E (ApoE) concentrations in mouse and human plasma were assessed using ELISA kits (MBS2512274) from MyBioSources (San Diego, CA) and Abcam (Ab108813, Cambridge, MA), respectively.

#### Lipoprint analysis

LDL and HDL subfraction distributions were analyzed with the Quantimetrix Lipoprint system (Quantimetrix Corporation, Redondo Beach, CA) according to the manufacturer’s instructions and as described elsewhere^28^. The system uses 25 μl of fasting plasma samples (not previously frozen). Up to 7 LDL bands can be detected. The LDL-1 and -2 bands correspond to large particles, whereas bands LDL-3 to 7 correspond to small dense LDL particles. Similarly, 10 HDL sub-fractions are detected. HDL-1 to -3 represent large particles, sub-fractions − 4 to − 7 are reported as medium particles, and sub-fractions − 8 to − 10 as small particles. This system also calculates the cholesterol concentration for each lipoprotein fraction according to a total cholesterol value obtained for each plasma sample.

### Macrophage isolation and differentiation

Bone marrow-derived macrophages were isolated and cultured as follows. Briefly, 3-month-old mice were euthanized by carbon dioxide inhalation. Hind legs were exposed and the muscle tissue removed to isolate the femurs and tibiae. The bone marrow was flushed from femurs and tibiae with culture medium (DMEM) containing 10% fetal calf serum and 1% penicillin/streptomycin/l-glutamine (Invitrogen, Carlsbad, CA) using a syringe and a 25-gauge needle. The cell suspension was drawn through a syringe with an 18-gauge needle to dissociate cell clumps and was passed through a 70-μm pore cell strainer (BD BioSciences, San Jose, CA) to remove tissue debris. The cells were plated and incubated for 5 days in the presence of 25 ng/ml macrophage colony stimulating factor (M-CSF) (PeproTech, Rocky Hill, NJ) in order to initiate macrophage differentiation. Foam cell differentiation from macrophages was achieved with 50 μg/ml acetylated LDL (Alfa Aesar, J65029) added to the cell culture media for 48 h. For macrophage polarization towards the classic M1 and alternate M2 phenotypes, macrophages were kept for 24 h in DMEM medium supplemented with 20 ng/mL TNFα for M1 differentiation and with 20 ng/mL IL-4 for M2 differentiation. Cells were harvested after 24 h for total RNA isolation.

### Cholesterol efflux

Bone marrow-derived macrophages (BMDMs) were seeded in 24-well plates at a density of 5 × 10^5^ cells per well in triplicate and allowed to adhere overnight. Cells were incubated with loading medium (DMEM/F12 Glutamax (Invitrogen), 1% penicillin/streptomycin, 0.2% fatty acid-free bovine serum albumin, 50 μg/mL acetylated LDL and 1 μCi/ml [^3^H]-cholesterol (NEN Life Science products, Boston, MA) for 36 h. The cells were then washed twice with PBS and equilibrated in 0.2% BSA in DMEM/F12 media for 2 h. Efflux media (DMEM, 0.2% BSA) with or without 20 μg/mL apoA1 (acceptor for ABCA1-mediated efflux) or 50 μg/mL HDL (acceptor for ABCG1-mediated efflux), was then added to each well for 7 h. Supernatants and cell lysates were then collected, and the amount of [^3^H]-cholesterol was determined with a scintillation counter. The percent cholesterol efflux at baseline or to ApoA1 or HDL was expressed as a percentage of the total loaded [^3^H]-cholesterol using the following equation: (total effluxed [^3^H]-cholesterol in media)/(total effluxed + total cellular [^3^H]-cholesterol).

### Immunohistochemistry on macrophages

Bone marrow-derived macrophages were seeded in 24-well plates onto small coverslips placed inside the wells. Cells were cultured as described above with or without 50 μg/mL acetylated LDL (for foam cell differentiation). Cells were then fixed and permeabilized with cold methanol for 5 min at − 20 °C. Rabbit monoclonal anti-ABCA1 (NB400-105SS, Novus Biologicals, Littleton, CO) and anti-ABCG1 (PA5-13462, Thermo Fisher, Waltham, MA) antibodies were used to specifically detect ABCA1 and ABCG1 in BMDMs. The secondary antibody was AlexaFluor 488 (Life Technologies, CA). The coverslips with the cells from the wells were placed onto slides and immunofluorescent images were acquired using an Axioskop 2 fluorescent microscope (Zeiss, Thornwood, NY). Individual images were collected and processed with Photoshop CS6 (Adobe, San Jose, CA).

### Western blot

ABCA1 and ABCG1 protein expression in bone marrow-derived macrophages and foam cells were also detected using standard western blot techniques we previously described^18^. Proteins were extracted on ice in RIPA buffer containing 1X protease inhibitor mini complete cocktail (Roche Applied Science, Indianapolis, IN), and 5 mmol/L EDTA. Homogenates were centrifuged at 16,000 *g* and the protein concentration in the supernatant was determined by absorbance using a Nanodrop spectrophotometer (ThermoScientific, Wilmington, DE). 50 μg was combined with reducing Novex sample buffer (Invitrogen, Carlsbad, CA) and loaded into wells of a 4–12% polyacrylamide gel (Invitrogen, Carlsbad, CA). Proteins were transferred to nitrocellulose membrane and blotted with the anti-ABCA1 and ABCG1 antibodies (NB400-105SS, Novus Biologicals, Littleton, CO and PA5-13462, Thermo Fisher, Waltham, MA). Appropriate secondary antibodies were detected using the Odyssey infrared imaging system (Li-cor, Lincoln, NE). β-actin was used as a loading control (Abcam Ab8229, Cambridge MA) and mouse lung lysates were used as a positive control (not shown).

### Reverse transcription PCR

Real-Time PCR was used to determine the level of mRNA expression of various genes in cultured macrophages and mouse tissues. Total RNA was extracted from macrophages/foam cells or approximately 20 mg of tissue samples using the RNeasy kit (Qiagen Inc., Valencia, VA). RNA was converted into first-strand cDNA using a SuperScript III First-Strand Synthesis SuperMix kit (Thermofisher, Waltham MA). The level of gene expression was detected by quantitative RT-PCR (qRT-PCR) with a StepOnePlus Real-Time System (Applied Biosystems, Foster City, CA) using commercially available TaqMan probes from Life Technologies. (Ccl2: Mm00441242_m1, TNFα: Mm00443258_m1, ApoE: Mm01307193_g1, Ccr2: Mm99999051_gH, CD36: Mm00432403_m1, Abca1: Mm00442646_m1, Abcg1: Mm00437390_m1, Hmgcr: Mm01282499_m1, SR-B1: Mm00450234_m1, Cyp7a1: Mm00484150_m1, ApoA1: Mm00437569_m1). As an endogenous control, gene *Hmbs* (Mm01143545_m1) was used for the macrophage/foam cell samples and *Gapdh* (Mm99999915_g1) was used for the liver samples.

### Plasma biliary acids measurement by UPLC-MS/MS

Bile acid measurements were performed with 50 μl of mouse plasma using D4-bile acids as internal standards. Each sample was deproteinized with 200 μl of acetonitrile (ACN). After a 10.000 × g centrifugation at 15 °C for 15 min, the upper phase was collected and dried under nitrogen at 50 °C. All samples were resuspended in 100 μl of a mixture (70:30, A:B, v:v) of a solvent A (95% ultrapure H_2_O / 5% acetonitrile (ACN) + 10 mM of ammonium formate) and B (20% ultrapure H_2_O / 80% ACN + 10 mM of ammonium formate). UPLC-MS/MS measurements were performed on a Waters Acquity H-class UPLCTM associated with a Waters Xevo triple quadripole equipped with an electrospray ionization (ESI) source. The UPLC was fitted with a Cortec UPLC C18 column (1.6 μm—2.1 Å ~ 100 mm; Waters) set at 40 °C and 10 μl of sample were injected onto the column. Elution was achieved with a linear gradient of solvent B in solvent A at a flow rate of 0.3 ml/min. The elution method was as follows: 10% of solvent B up to 45% in 8 min—45% of solvent B up to 90% of solvent B in 4 min—90% of solvent B kept constant for 1 min—90% of solvent B down to 10% of solvent B in 1 min—10% of solvent B kept constant for 1 min prior to the next injection. ESI was used in negative mode with a capillary, cone and collision voltages set at 3 kV, 80 V and 12 eV, respectively. All UPLCMS/MS data were integrated and analyzed with MassLynx and TargetLynx softwares (Waters Corp. Milford, MA).

### Cytokine and Chemokines

TNFα, IFNγ, IL-1 β, IL-6, IL-10, IL-12p70, IL-17A, CCL2, CCL4, CCL5, CXCL8 and CXCL10 concentrations were measured in human plasma samples by multiplex fluorescent-bead-based technology (Luminex 200, Austin, TX) using a commercially available Luminex screening assay (R&D Systems, Minneapolis, MN). Cytokines/chemokines were measured in mouse plasma samples using the Th1 / Th2 / Th17 Cytokines Multi-Analyte ELISArray Kits from QIAGEN Inc. (Valencia, VA).

### Data analysis

Results are presented as the mean +/− standard error of the mean (SEM). Animal numbers used for individual sets of data varied between experiments and are shown on the figures. For multigroup comparisons, one-way ANOVA (F) followed by the Tukey HSD-test was used for detecting the statistical differences. The ANOVA F values shown in the results are the ratio of two mean square values. If the null hypothesis is true, F is expected to have a value close to 1.0. A large F ratio signifies that the variation among group means is more than you'd expect to see by chance. The Student’s *t*-test was used for detecting differences between two groups by comparing the effect of one variable such as genotype, cell differentiation or diet. Relative gene expression was calculated using the delta-delta threshold method^29^. Difference between relative gene expressions greater than 1.5-fold were considered statistically significant. Results from the comparison between PXE patients and the NUMEVOX cohort (described in the results section and Table 2) were analyzed using Kruskal Wallis and performed with Stata 12.0 software (StataCorp, Texas, USA). The level of statistical significance was set at *P* < 0.05. Statistical comparison into the groups were performed using Wilcoxon’s test. A *p* value < 0.05 was considered statistically significant. Analyses were performed using GraphPad’s PRISM software v.8.4.2 (San Diego, CA).

## Results

### ABCC6 deficiency induces dyslipidemia in mice and humans

The premise of this study was that ABCC6 may influence the levels of plasma lipoproteins and susceptibility to atherosclerosis. To explore this possibility, we generated 3 mouse genotypes with normal, reduced and no *Abcc6* expression in an atherogenic background (*Ldlr*^−/−^) to determine if gene dosage affects dyslipidemia, atherosclerosis and possibly vascular calcification.

#### Mice

We first determined the effect of ABCC6 deficiency on lipoprotein profiles using a Lipoprint analyzer in several mouse models fed either normal chow or a high fat diet for 16 weeks. We used *Abcc6*^+*/−*^;*Ldlr*^−/−^ and *Abcc6*^−/−^*;Ldlr*^−/−^ mice in comparison with *Ldlr*^−/−^ controls as well as *Abcc6*^−/−^ and wild type (WT) animals. The results, summarized in Table 1, showed that under non-atherogenic conditions (*i.e.* normal diet and *Ldlr*^+*/*+^ status), ABCC6 deficiency leads to substantially reduced HDL and increased LDL levels (*p* = 0.03 and *p* = 0.0035, n = 8 *versus* n = 3, respectively). Lipoprint analysis allowed the quantitative evaluation of the main HDL and LDL sub-fractions and notably revealed that the large HDL subclass, which shows an inverse relationship to cardiovascular risk, was significantly reduced in *Abcc6*^−/−^ mice (Supplemental Table 1). Interestingly, the highly atherogenic sub-fractions LDL 3 through 7 (labelled as “Large LDL” on Supplemental Table 1) were elevated in *Abcc6*^−/−^ mice but only in animals fed normal chow. When compared to control mice, total cholesterol and HDL levels were particularly affected in *Abcc6*^−/−^ mice fed a lipid-rich diet, though no atherosclerosis was noted in these animals (not shown). Triglyceride levels were significantly higher in all *Abcc6*-deficient animals as compared to *Ldlr*^−/−^ or WT controls, except for the double KO on high fat diet (Table 1). Under severe atherogenic conditions (*Ldlr*^−/−^ and high fat diet), experimental mice displayed a ~ tenfold increase in total cholesterol as compared to *Ldlr*^+*/*+^ mice but there was no significant difference between the three genotypes. However, major alterations of HDL and LDL levels were found in both *Abcc6*^+*/−*^;*Ldlr*^−/−^ and *Abcc6*^−/−^*;Ldlr*^−/−^ mice in comparison to *Ldlr*^−/−^ controls (Table 1). The gradual decrease to undetectable levels of the small HDL fraction was notable (Supplemental Table 1).

**Table 1:** Lipoprotein profile analysis of mouse models and human subjects lacking ABCC6.

Diets	Genotype		Total	LDL	HDL	Triglycerides
	*Ldlr*	*Abcc6*	**Cholesterol**			
			mg/dL	mg/dL	mg/dL	mg/dL
High fat	−/−	+/+	1354 ± 92.8	534.6 ± 37.1	241.2 ± 33.2	74.47 ± 15.1
High fat	−/−	+/-	1175 ± 84.3	811.2 ± 56.2**	134.3 ± 31.3*	64.51 ± 10.2
High fat	−/−	−/−	1318 ± 47.3	900.3 ± 49.6***	101.8 ± 8.2**	67.54 ± 9.26
Chow	−/−	+/+	234.8 ± 35.4	158.0 ± 33.0	59.8 ± 5.4	6.12 ± 2.3
Chow	−/−	+/-	310.7 ± 25.9	130.0 ± 17.9	56.6 ± 12.2	11.74 ± 2.8**
Chow	−/−	−/−	255.0 ± 26.9	165.8 ± 14.9	70.2 ± 12.8	26.31 ± 3.5***
High fat	+/+	+/+	168.8 ± 18.0	37.6 ± 8.8	115.8 ± 5.3	3.52 ± 0.25
High fat	+/+	−/−	121.7 ± 3.5**	42.5 ± 3.5	**68.6 ± 4.2**^**####**^	5.97 ± 1.34*
Chow	+/+	+/+	131.7 ± 9.6	25.7 ± 5.4	101.0 ± 10.7	2.24 ± 0.21
Chow	+/+	−/−	125.4 ± 4.6	41.6 ± 1.6**	**76.2 ± 4.6**^**#**^	3.61 ± 0.89*
Healthy controls			204.6 ± 12.3	121.6 ± 8.2	68.5 ± 5.1	85.22 ± 8.4
PXE patients			188.6 ± 7.6	113.0 ± 5.8	**50.5 ± 2.2**^**###**^	102.2 ± 8.9

**Bold** shows significant differences with controls.

Mice age: 5 months-old, n = 3 to 8.

Humans**:** Age- and sex-matched: PXE: 45.2 ± 2.5 years; Controls: 45.3 ± 2.7 years, Ctrl = 14, PXE = 32.

*p*-values were determined by one-way ANOVA and Tukey’s post hoc test (*) or Student’s *t*-test (#).

*, ^#^ *p* < 0.05, **, ^##^ *p* < 0.01, ***, ^###^ *p* < 0.001 ****, ^**####**^ *p* < 0.0001.

Because ABCC6 deficiency did not affect atherosclerosis development in mice with an *ApoE*^−/−^ background as reported^30^, we specifically examined the plasma levels of this apolipoprotein in *Abcc6*^+*/−*^;*Ldlr*^−/−^ and *Abcc6*^−/−^*;Ldlr*^−/−^ mice, as it may have been a contributor to the enhanced atherosclerosis phenotype we observed. However, there was no significant variation as compared to *Ldlr*^−/−^ controls (Supplemental Fig. 1A).

**Figure 1:**
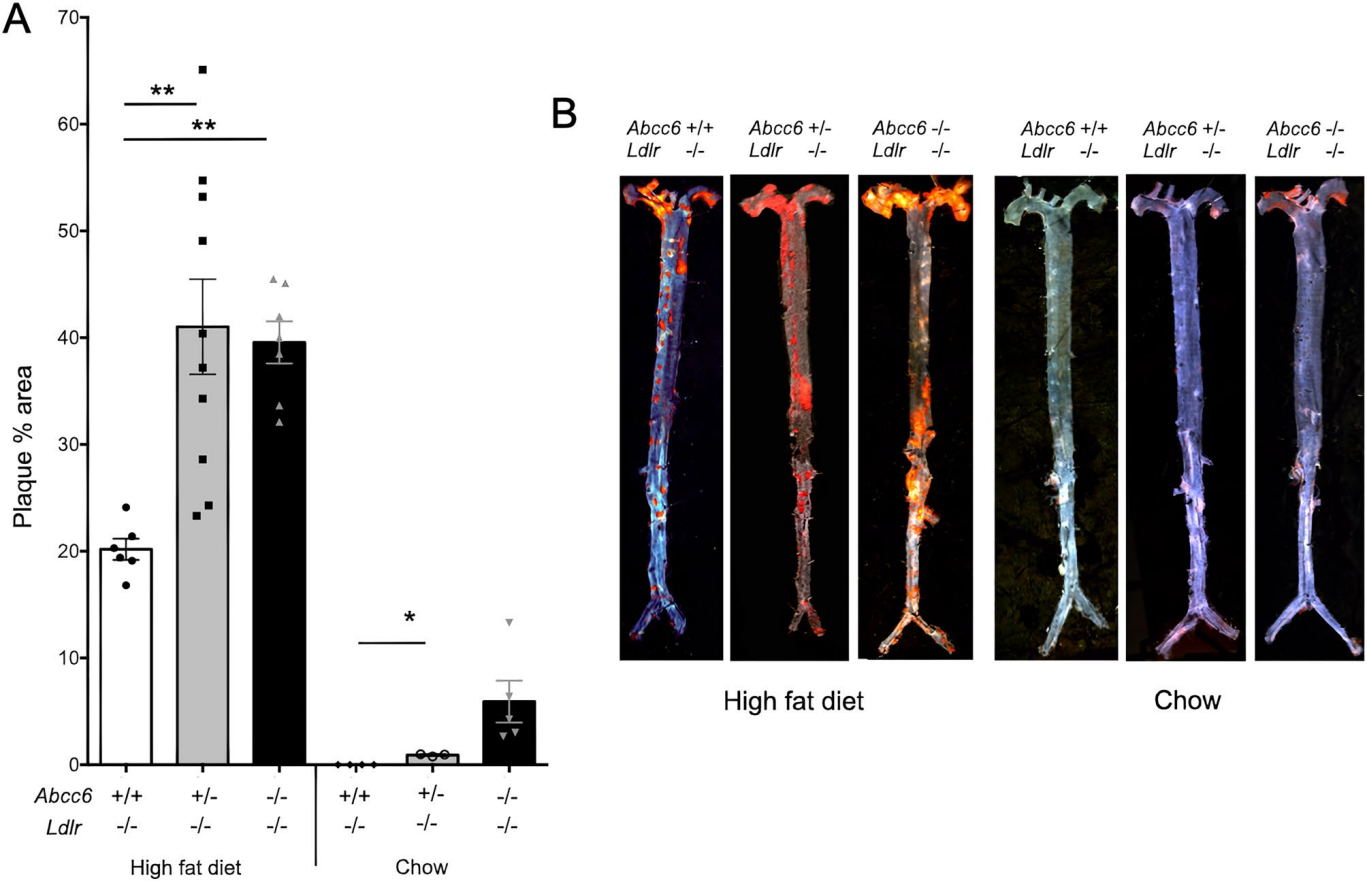
Atherosclerosis development in *Ldlr*^−/−^ mice lacking *Abcc6*. Atherosclerotic plaque deposition in aorta was assessed by measuring the *en face* surface area stained by Oil Red O staining. Littermate mice were maintained on an atherogenic diet (high fat diet) or regular chow for 16 weeks. (**A**) Quantification of aortic lesions is shown as percentage of the aorta surface. (**B**) Representative images of the Oil Red O staining. Results are shown as means ± SEM. *p*-values were determined by one-way ANOVA and Tukey’s post hoc test. *p < 0.05, **p < 0.01, ****p < 0.0001.

#### PXE patients

We followed up the murine investigation with a comprehensive analysis of the lipoproteins from PXE patients (Table 1). Total cholesterol levels were not statistically significant from healthy age- and sex-matched controls. However, the patients presented a highly significant reduction in HDL levels (− 24%, *p* = 0.0004) in the same proportion as *Abcc6*^−/−^ *vs* wild type mice on a normal diet. The small and large HDL sub-fractions were also decreased as compared to healthy controls (Supplemental Table 1) but there was no change in LDL, unlike the animal models. We found no difference in plasma triglyceride levels (Table 1) or plasma ApoE concentration (Supplemental Fig. 1B) overall between PXE patients and healthy controls. However, significant differences in triglyceride levels were found between sub-groups. Triglycerides were elevated in control males (+ 82%, but still within normal range) as compared to control females (*p* = 0.0013, n = 10 *versus* n = 24). Average plasma triglyceride levels were also higher (+ 47%) in female patients *vs* female controls (*p* = 0.0066, n = 24), but, remarkably, there was no difference between male and female PXE patients (*p* = 0.88).

### The lack of ABCC6 enhances atherosclerosis

#### Mice

**Figure 2:**
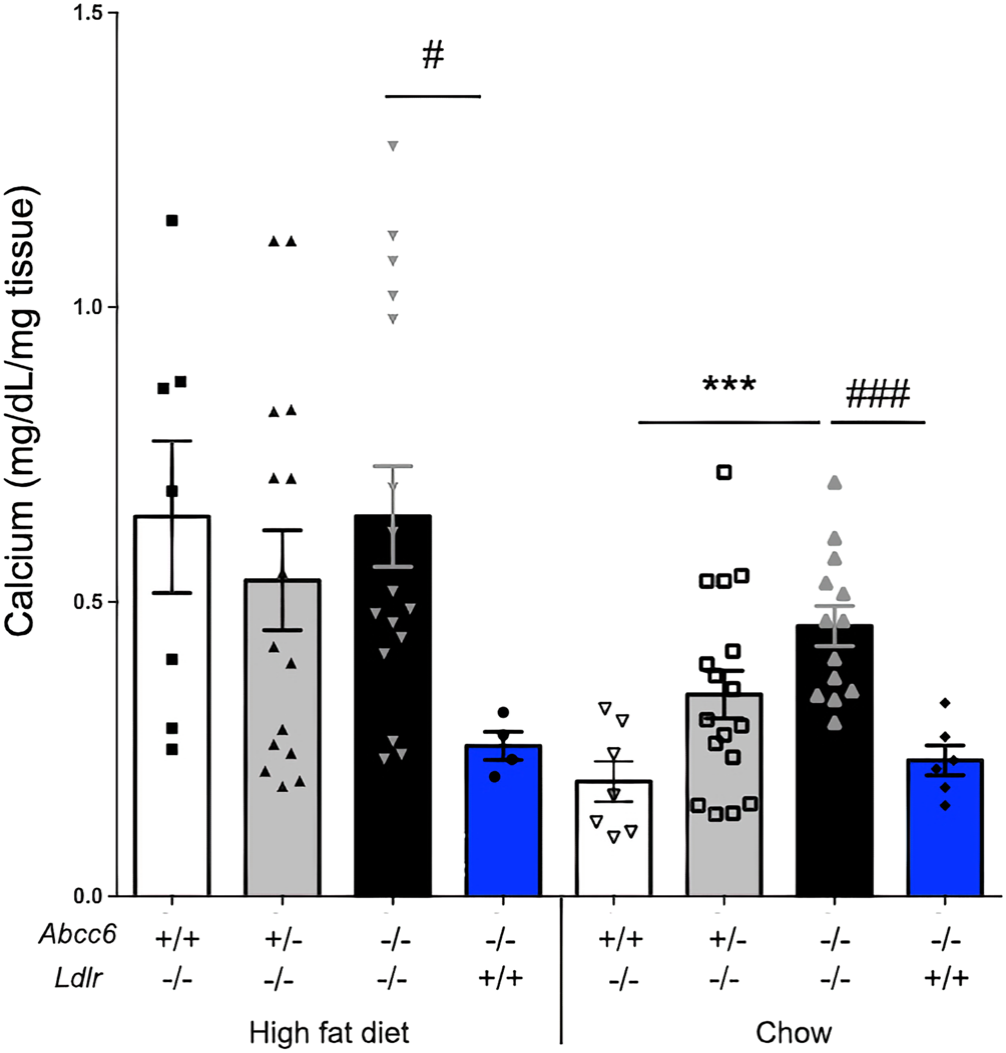
Aortic calcification in *Ldlr*^−/−^ mice lacking *Abcc6*. The level of calcification in the aorta was measured by total calcium content of the full aorta normalized to the weight of the tissue. Littermate mice were maintained on an atherogenic diet (high fat) or regular chow for 16 weeks. As shown by the blue bars, the level of vascular calcification induced by *Abcc6*-deficiency was not influenced by the diet. Results are shown as means ± SEM. *p*-values were determined by one-way ANOVA and Tukey’s post hoc test (*) or Student’s *t*-test (#).*, ^#^*p* < 0.05, **p < 0.01, ***, ^###^*p* < 0.001.

*Ldlr*^−/−^, *Abcc6*^+*/−*^;*Ldlr*^−/−^ and *Abcc6*^−/−^*;Ldlr*^−/−^ mice were subjected to 16 weeks of high fat diet to induce atherosclerotic lesions. Quantification of aortic plaque using the *en face* method from the three strains of mice revealed a statistically significant increase in plaque development in mice lacking ABCC6 as determined by one-way ANOVA *F* (2, 20) = 9.205 *p* = 0.0015. Particularly noteworthy, plaque development in heterozygous *Abcc6*^+*/−*^;*Ldlr*^−/−^ mice was as pronounced as it was in *Abcc6*^−/−^*;Ldlr*^−/−^ mice (*p* = 0.80) despite the presence of a functional *Abcc6* allele (Fig. 1). Under normal chow conditions, we did not observe any significant atherosclerosis in *Ldlr*^−/−^ mice, as expected; however, *Abcc6*^+*/−*^;*Ldlr*^−/−^ and *Abcc6*^−/−^*;Ldlr*^−/−^ mice presented small but clear evidence of statistically significant lipid deposition in the aortic arch (one-way ANOVA *F* (2, 9) = 5.338 *p* = 0.0296) that mirrored calcification increases under the same conditions (Fig. 2).

As the highest level of *Abcc6* expression is normally found in the liver^12^, we verified whether the lack of hepatic ABCC6 function could affect the expression of selected genes involved in reverse cholesterol transport (*Abca1*, *Abcg1*, *Sr-b1, ApoA1*) or cholesterol metabolism (3-hydroxy-3-methylglutaryl CoA reductase or *Hmgcr and Cyp7*α*1*). There was no significant change in *Abcc6*^−/−^*;Ldlr*^−/−^ mice as compared to control *Ldlr*^−/−^ mice fed normal or high fat diets except in *Abcg1* expression which was significantly downregulated (*p* = 0.02) under high fat dietary conditions in an *Abcc6*-dependent manner (Supplemental Fig. 2B).

#### PXE patients

To determine if PXE patients are susceptible to vascular remodeling, we performed a retrospective analysis of data collected from 142 patients routinely followed at the University Hospital of Angers, France (by L.M. and G.L.). The effect of ABCC6 deficiency on the vasculature has already been extensively documented in many comparisons with healthy controls^22,31–35^. Therefore, to provide a novel disease perspective to which the effects of the PXE pathophysiology could be gauged against, we compared data from PXE patients with a cohort already at risk for significant cardiovascular anomaly *i.e.* 668 dysmetabolic individuals screened from the NUMEVOX cohort^36^.

In both cohorts, carotid intima-media thickness (IMT) was determined using ultrasound imaging coupled with echotracking according to standardized methods while plaques were scored (*i.e.* presence/absence) bilaterally at the carotid, abdominal aorta and femoral bifurcations. Note that in the absence of a direct histopathology analysis, the nature of the plaque seen in PXE patients and control individuals is herein assumed to be of atheromatous nature. PXE patients showed no significant difference in intima-media thickness (IMT) as compared to the cohort at risk (617 ± 164 µm *versus* 650 ± 121 µm, *p* = 0.720). Adjustment for age and number of sites with atherosclerotic plaques did not affect the lack of statistical significance (β = − 0.0011, *p* = 0.312). However, PXE patients who had different (better) lipid profiles with modest dyslipidemia (average cholesterol ratio of 3.7) presented a higher number of atherosclerotic sites as compared to the NUMEVOX cohort (1 ± 3 *versus* 2 ± 4, *p* = 0.023). After adjusting for age, PXE patients still showed a significantly larger number of atherosclerotic sites than dysmetabolic controls (β = 0.39, *p* = 0.015), despite having less arteriosclerosis. These data (Table 2) suggest that atherosclerotic plaques at typical sites are more prevalent in PXE patients than in subjects with metabolic syndrome who are already at an increased risk for cardiovascular diseases. This suggests that the disease status of PXE patients enhances susceptibility to plaque formation.

**Table 2:** Logistic regression between disease status and age, number of atherosclerotic plaque sites and intima media thickness (IMT).

	Model	
	β	SEM
Age (years)	− 0.074***	0.01
Number of sites with plaques	0.159*	0.069
IMT (µm)	0.000	0.001
Constant	1.822***	0.552

β = slope coefficient, SEM = standard error of the mean.

Constant = predicted value when all the X variables = 0.

*p*-values were determined using Wilcoxon’s test.

*p < 0.05, ***p < 0.0001.

### Enhanced systemic inflammation is associated with ABCC6 dysfunction

#### Abcc6-deficient mice

It is now well accepted that atherosclerosis is a chronic inflammatory disease mediated by the concerted action of many circulating pro-inflammatory cytokines. Therefore, we measured selected cytokines in the plasma of *Ldlr*^−/−^ and *Abcc6*^−/−^*;Ldlr*^−/−^ mice fed regular chow. We found no difference or no detectable levels for IL-2, IL-4, IL-13, IL-23, TNFα, TGFβ1 or IFNγ, in both strains of mice. However, significant levels of the pro-inflammatory cytokines IL-6, IL-12 and IL17A were measured in the plasma of *Abcc6*^−/−^*;Ldlr*^−/−^ mice (27.3 ± 1.9 pg/mL, 0.50 ± 0.04 pg/mL, 4.1 ± 0.6 pg/mL, n = 3, respectively) but were undetectable in *Ldlr*^−/−^ controls.

#### PXE patients

We also profiled cytokines and additional chemokines in the plasma of PXE patients in comparison to healthy controls. There was no difference in the levels of the pro-inflammatory cytokines TNFα, IL-1β, IL-17A, CCL2, CCL4, CXCL8 and CXCL10, the anti-inflammatory cytokine IL-10 or the Th1-related cytokines IFNγ and IL-12. Remarkably, PXE patients showed elevated plasma levels of pro-inflammatory (atherogenic) cytokine IL-6 (1.11 ± 0.35 pg/mL, n = 31 *versus* 0.24 ± 0.08, n = 24; *p* = 0.03) as well as the pro-atherogenic chemokine CCL-5 (24,781 ± 3240, n = 31 *versus* 6374 ± 907, n = 24; *p* < 0.0001). Of note, because values for IL-6 obtained with the Luminex system were at the limit of detectability, plasma values were verified with a Quantikine HS ELISA Kit (R&D Systems, Minneapolis, MN) and similar values were obtained (1.91 ± 0.41 pg/mL, n = 31 *versus* 0.81 ± 0.09, n = 25; *p* = 0.023).

### ABCC6 deficiency does not influence vascular calcification associated with atherosclerosis

#### Mice

After 16 weeks of high fat or normal chow feeding, we found elevated but equivalent levels of overall aortic calcification (2.7 fold, *p* < 0.05) in all three *Ldlr*^−/−^, *Abcc6*^+*/−*^;*Ldlr*^−/−^ and *Abcc6*^−/−^*;Ldlr*^−/−^ genotypes fed a high fat diet as compared to *Abcc6*^−/−^ mice under the same conditions (Fig. 2). This result suggested that the increased calcification was only a consequence of atherosclerotic plaque (intimal) and not linked to the *Abcc6* genotype. This conclusion is supported by the increased mineralization in *Abcc6*^+*/−*^;*Ldlr*^−/−^ and *Abcc6*^−/−^*;Ldlr*^−/−^ mice fed regular chow when compared to control *Ldlr*^−/−^ and *Abcc6*^−/−^ mice, as the first two mouse models presented atherosclerotic lesions whereas the two controls did not (Fig. 2).

For control purposes, we evaluated whether the absence of the *Ldlr* gene and a lipid-rich diet would alter the typical PXE calcification phenotype found in *Abcc6*^−/−^ mice. The lack of *Ldlr* alone or in combination with high fat diet had no significant influence on the mineralization of the vibrissae capsule (Supplemental Fig. 3A). High fat diet can also induce cardiac calcification in mice lacking functional ABCC6^20^. Here, there was no evidence of high fat diet-related DCC in the relatively short timeframe (16 weeks) of this study (Supplemental Fig. 3B).

**Figure 3:**
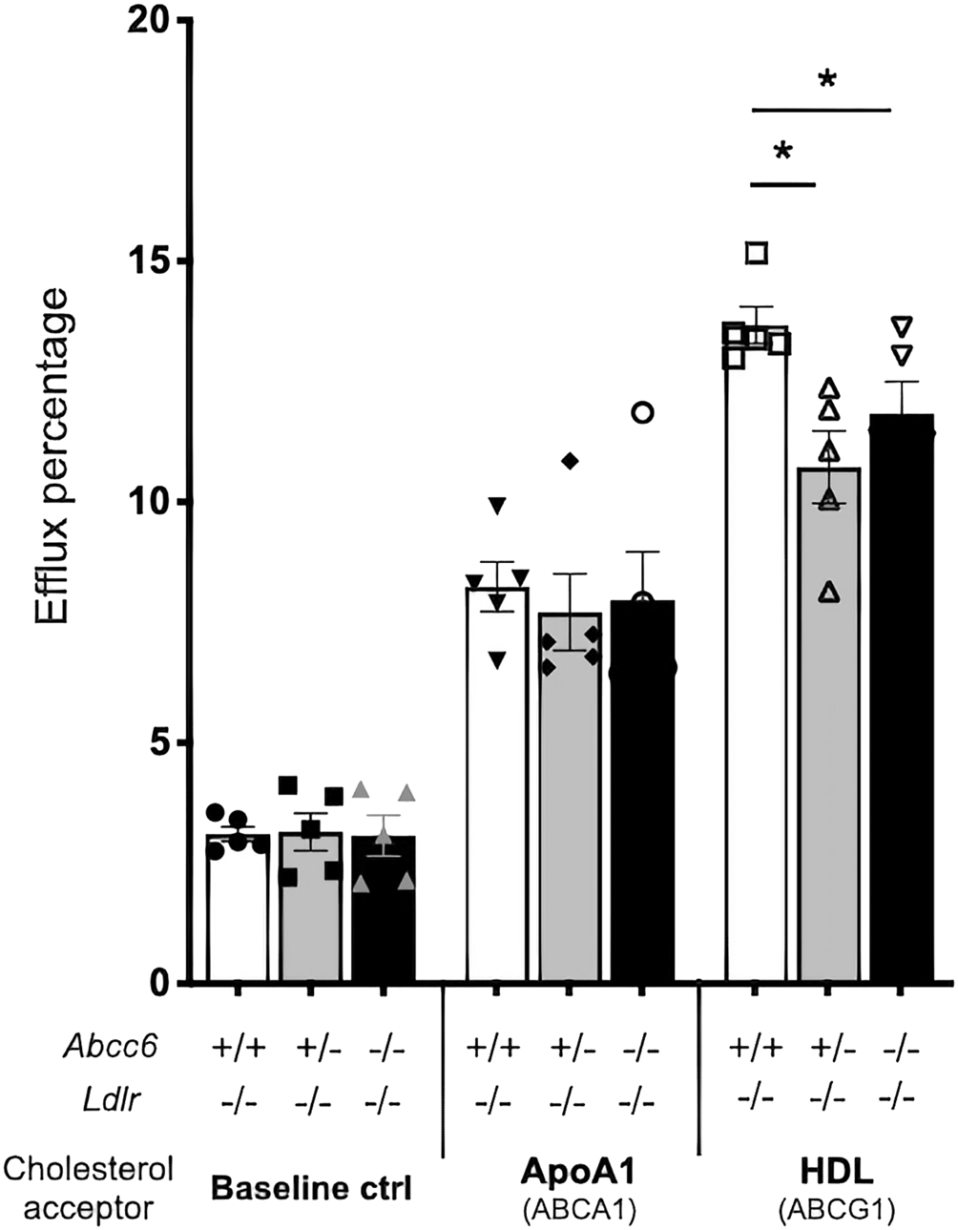
Cholesterol efflux from isolated bone marrow-derived macrophages. Macrophages were pre-loaded with 1 μCi/mL of cholesterol and 50 μg/mL AcLDL in 24-well plates using media with 10% lipoprotein deficient serum for 36 h. Lipid-free ApoA1 or HDL were used as cholesterol acceptors in a 7-h incubation period. The mouse genotypes are indicated. Results are shown as means ± SEM. *p*-values were determined by one-way ANOVA and Tukey’s post hoc test (*). *p < 0.05.

#### PXE patients

As skin calcification cannot be readily quantified in PXE patients, we determined arterial calcium score as previously described^34^. Not surprisingly, our cohort of patients displayed significantly elevated arterial calcium score as compared to healthy controls (TEP scan units 1356 ± 430.3, n = 16 *vs* 137.9 ± 68.3, n = 17 respectively, *p* = 0.007.), however, these measures did not readily distinguish medial from intimal calcification.

### The role of macrophages in dyslipidemia and atherosclerosis associated with ABCC6 deficiency in mice

Macrophages play a prominent role in the development of atherosclerotic lesions, and this has been extensively documented^37,38^. These immune cells normally efflux cholesterol to ApoA1, nascent and mature HDL via the ATP-binding cassette transporter A1 (ABCA1) and ABCG1, which are key steps in reverse cholesterol transport. The dysregulation of this process in vascular macrophages promotes atherogenesis^39^. To determine the role of macrophages in the dyslipidemia and atherosclerosis phenotypes we observed in ABCC6-deficient mice (and PXE patients), we first used bone marrow-derived macrophages isolated from our experimental mice to study cholesterol efflux. We found no difference of baseline efflux (without acceptor) or efflux towards ApoA1 between the three genotypes. However, when HDL particle acceptors were present, there was a small but significant decrease in efflux in equivalent proportions in *Abcc6*^+*/−*^;*Ldlr*^−/−^ and *Abcc6*^−/−^*;Ldlr*^−/−^ macrophages (Fig. 3).

**Figure 4:**
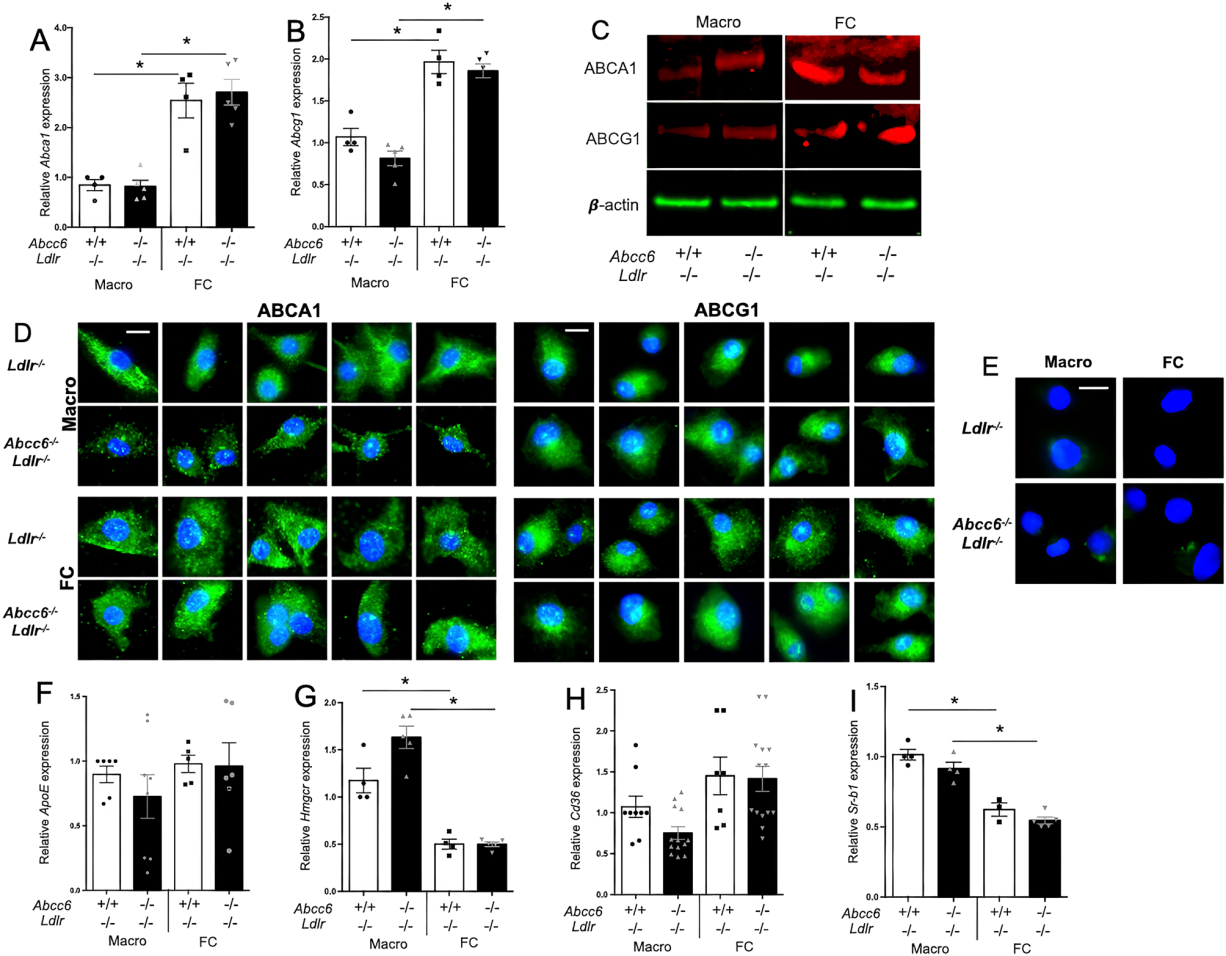
Expression of genes/proteins related to cholesterol efflux in bone marrow-derived macrophages and foam cells. Real-time RT-PCRs were performed using TaqMan probes specific for *Abca1* (**A**)*, Abcg1* (**B**)*, ApoE* (**F**), *Hmgcr* (**G**)*, Cd36* (**H**) and *Sr-b1* (**I**) cDNAs. Units are the relative gene expression normalized to *Hmbs*. Most results showed expected differences between macrophages and foam cells but not between the genotypes. Results are shown as means ± SEM. *p*-values were determined by Student’s *t*-test, * *p* < 0.05. (**C**) Representative western blot images showing the levels of both ABCA1 and ABCG1 expression (red signal) in foam cells (FC) as compared to macrophages (Macro). β-actin (green) served as loading control. Data points represent individual mice from which bone marrow-derived macrophages were isolated and used for experiments. (**D**) Immunofluorescent detection (green signal) of ABCA1 and ABCG1 on macrophages (Macro) and foam cells (FC) derived from *Abcc6*^−/−^;*Ldlr*^−/−^ and control *Ldlr*^−/−^ mice. Five representative images from each condition are shown. We only observed some staining pattern variation for ABCA1 in macrophages from *Abcc6*^−/−^;*Ldlr*^−/−^ mice which appeared punctated as compared to cells from control *Ldlr*^−/−^ mice. (**E**) Negative controls for the immunofluorescent staining shows the specificity of the primary antibodies used. Nuclei were stained with DAPI.

**Figure 5:**
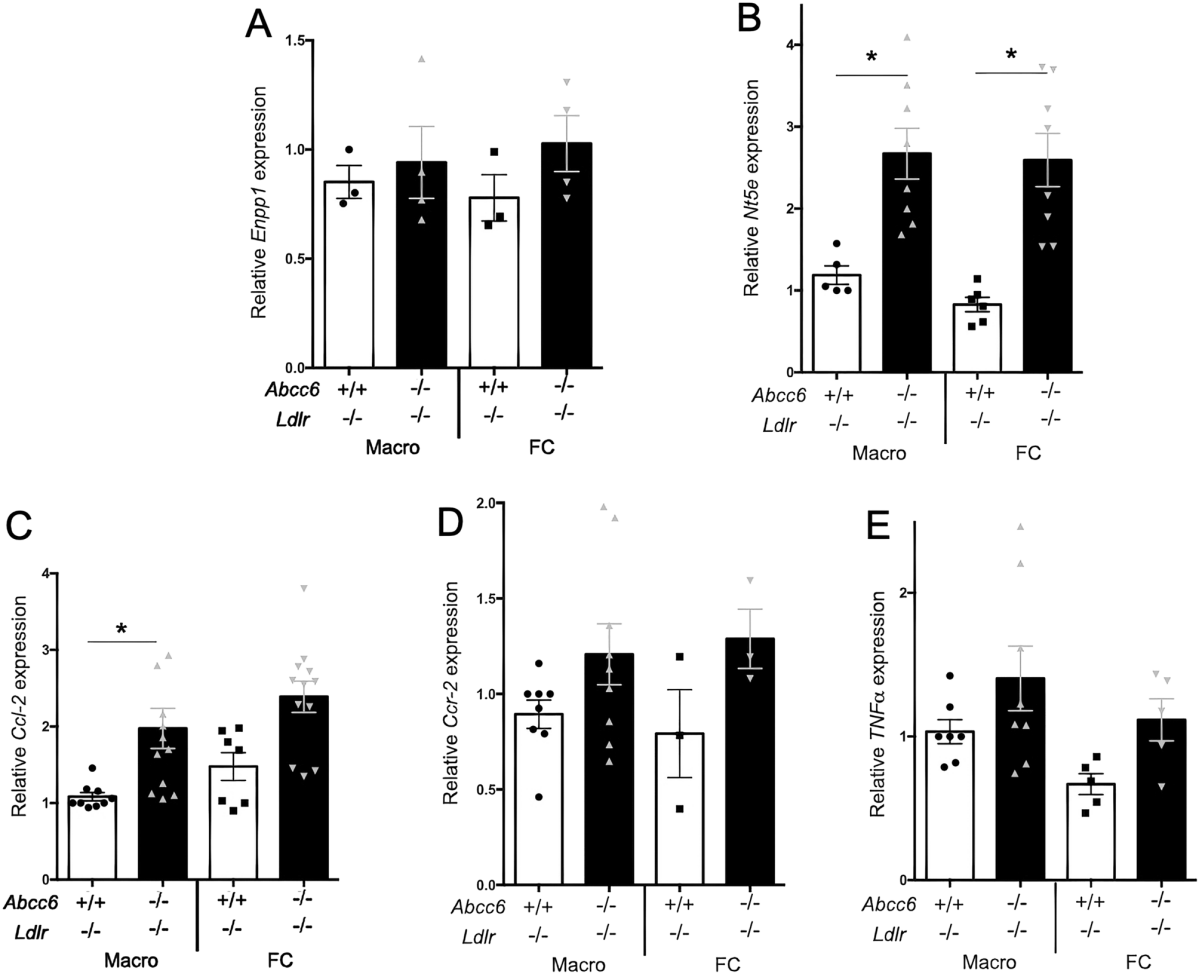
Expression of genes related to cholesterol and extracellular purine metabolisms and cytokines/chemokines in macrophages and foam cells derived from *Abcc6*^−/−^;*Ldlr*^−/−^ and control *Ldlr*^−/−^ mice. Real-time RT-PCRs were performed using TaqMan probes specific for *Enpp1* (**A**), *Nt5e* (**B**), *Ccl-2* (**C**)*, Ccr-2* (**D**) and *TNF-α* (**E**) cDNAs. Units are the relative gene expression normalized to *Hmbs*. The most consistent changes and probably the most physiologically relevant are shown in panels (**B**,**C**) with *Nt5 and Ccl-2* expression significantly increased in cells lacking *Abcc6*. Data points represent individual mice from which bone marrow-derived macrophages were isolated and used for experiments. Results are shown as means ± SEM. *p*-values were determined by Student’s *t*-test, *p < 0.05.

To investigate a possible cause of this decrease in efflux, we quantified the expression of *Abca1, Abcg1, ApoE, Hmgcr, Cd36 and Sr-b1* genes in both macrophages and foam cells from *Ldlr*^−/−^ and *Abcc6*^−/−^*;Ldlr*^−/−^ mice. The differentiation of macrophages into foam cells enhanced the expression of *Abca1* and *Abcg1,* but there was no *Abcc6*-specific effect (Fig. 4A,B). These results were confirmed at the protein level as well (Fig. 4C,D,E). Similarly, the expression of *Hmgcr and Sr-b1* dropped in foam cells but no *Abcc6*-dependent effect was observed (Fig. 4G,H,I). This result is somewhat similar to previous findings^40^. *ApoE* expression (Fig. 4F) showed no difference, which was consistent with plasma levels (Supplemental Fig. 1A), though *Cd36* showed a modest (but not significant) expression decrease in *Abcc6*^−/−^*;Ldlr*^−/−^ macrophages (Fig. 4H). Because the changes in gene expression were either small and/or inconsistent between macrophages and foam cells, they seemed unlikely to be physiologically relevant. The expression of *Enpp1* and *Nt5e* was also analyzed as these genes encode proteins immediately downstream of the pathway initiated by ABCC6^5, 9, 10^. There was no significant change for *Enpp1*, but the expression of *Nt5e* was upregulated in both macrophages and foam cells from *Abcc6*^−/−^*;Ldlr*^−/−^ mice (Fig. 5A,B). Furthermore, we analyzed the expression of selected cytokine genes that play a role in atherosclerosis. We only found a significant increase in the expression of *Ccl-2 (Mcp-1)* in *Ldlr*^−/−^;*Abcc6*^−/−^ macrophages *vs Ldlr*^−/−^ controls, Fig. 5C) whereas *Ccr-2* and *TNF-α* showed not significant change (Fig. 5D,E).

Remarkably, we found no significant *Abcc6* expression in bone marrow-derived macrophages M1 or M2 activated or foam cells from *Ldlr*^−/−^ mice (not shown).

### Changes in plasma bile acids in mice lacking ABCC6

**Figure 6:**
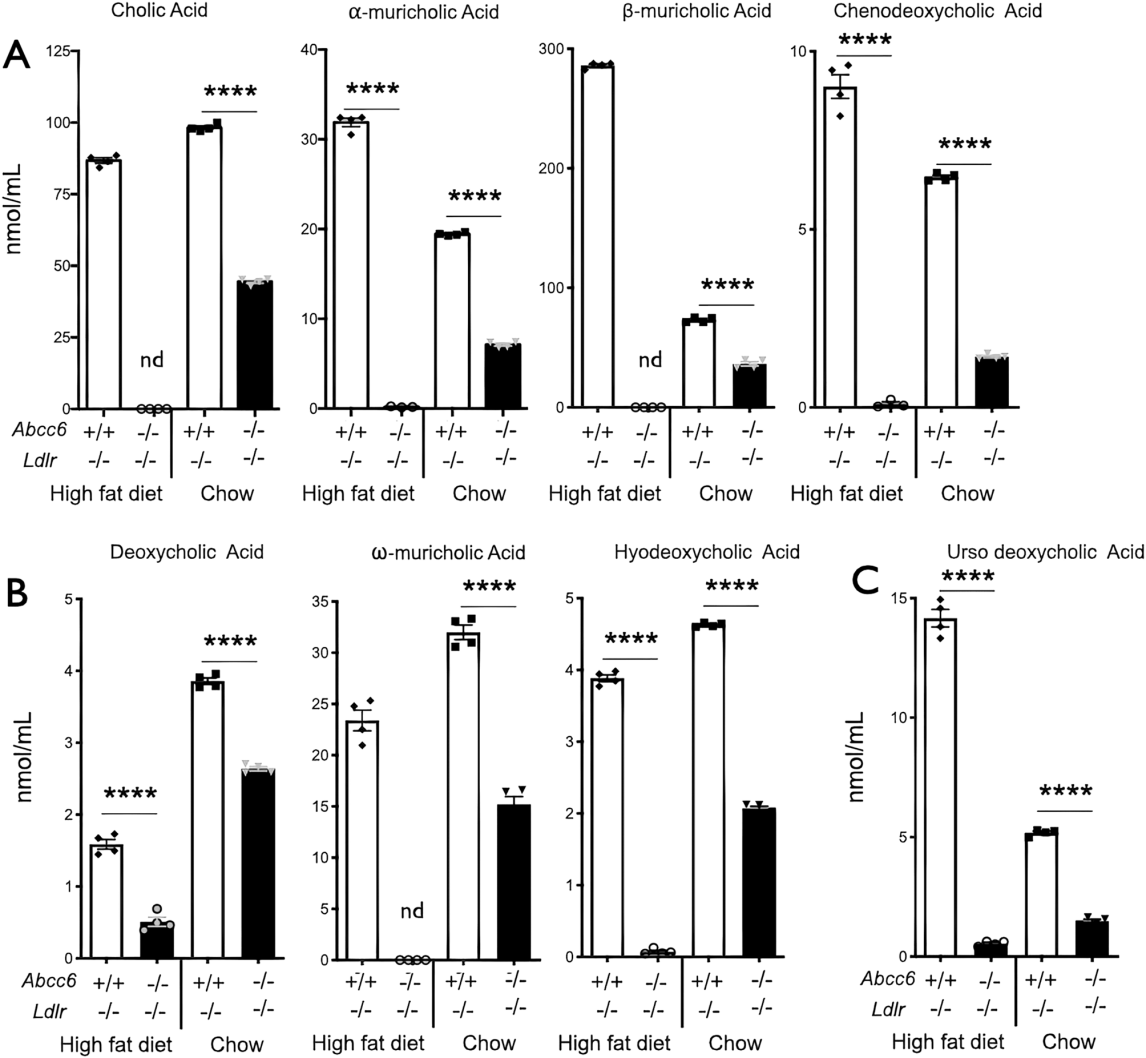
Plasma bile acid profiles in *Ldlr*^−/−^ mice lacking *Abcc6.* Littermate mice were maintained on an atherogenic diet (high fat) or regular chow for 16 weeks. Panel (**A**) shows the primary bile acids: cholic, α- and β-muricholic acids and chenodeoxycholic acid. Panel (**B**) illustrates results for the secondary bile acids deoxycholic acid, ω-muricholic acid and hyodeoxycholic acid. Panel (**C**) represents data for the tertiary ursodeoxycholic acid. Results are shown as means ± SEM. *p*-values were determined by Student’s *t*-test. ****p < 0.0001.

Cholesterol (as HDL) is brought to the liver via reverse transport for recycling or elimination. Because bile acids are quickly absorbed in the intestines and recycled towards the liver via the enterohepatic recirculation, we measured the levels of bile acids in the plasma of *Abcc6*^−/−^;*Ldlr*^−/−^ and control *Ldlr*^−/−^ mice fed either normal or high fat diets. In both dietary conditions, we found a profound decrease in the circulation of the following bile acid concentrations: cholic, α- and β-muricholic acids, chenodeoxycholic acid, deoxycholic acid, ω-muricholic acid, hyodeoxycholic acid and ursodeoxycholic acid. However, this decrease was more pronounced in mice fed high fat diet than normal chow (Fig. 6). Lithocholic acid was not detectable in our samples (not shown) but the other 8 primary, secondary and tertiary bile acids showed drastic reductions ranging from − 55 to − 99% (*p* < 0.0001) in *Abcc6*^−/−^;*Ldlr*^−/−^ mice with cholic, β-muricholic and ω-muricholic acids being the most affected, decreasing to undetectable levels.

**Figure 7:**
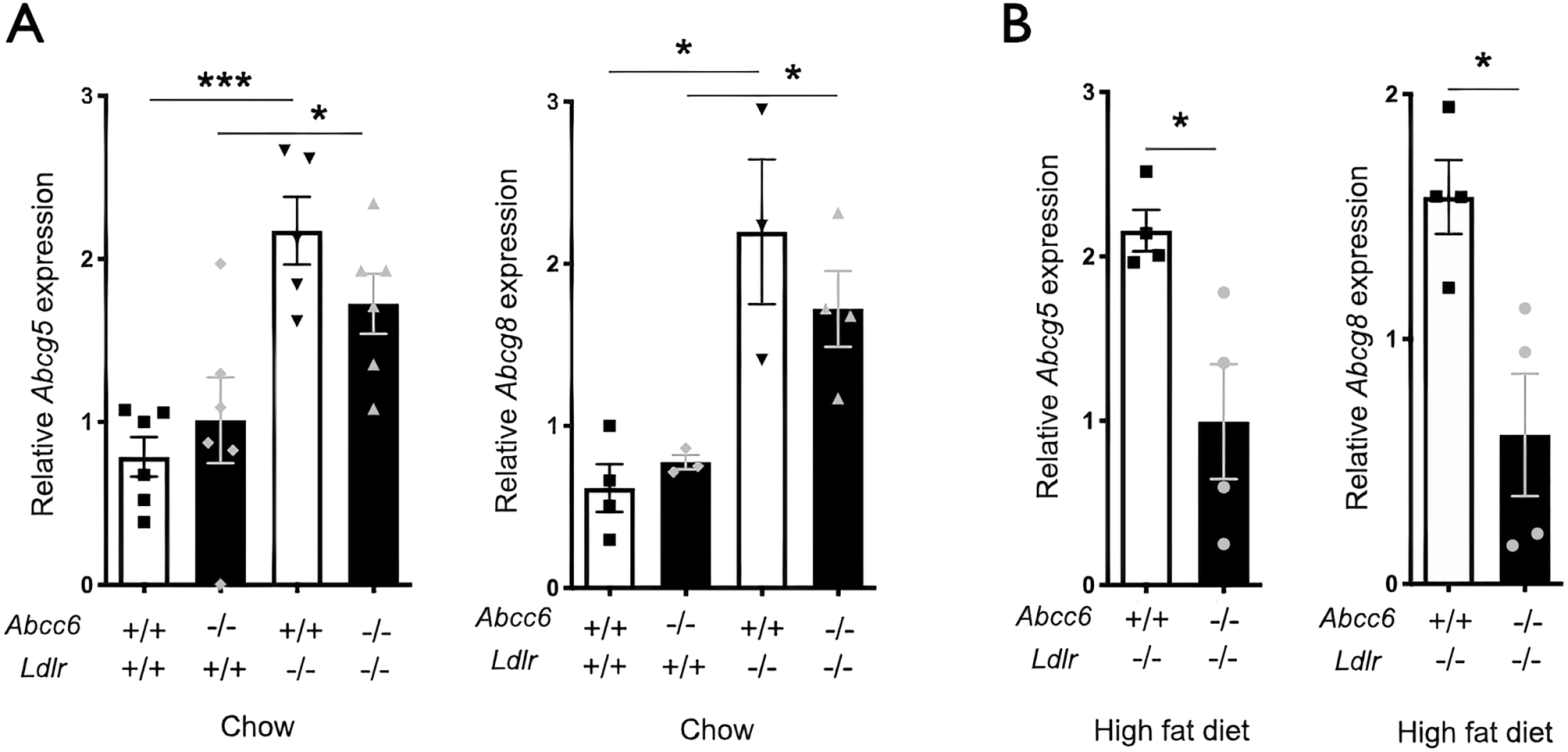
Expression of the *Abcg5* and *Abcg8* genes in the liver of *Abcc6*^−/−^;*Ldlr*^−/−^ and control *Ldlr*^−/−^ mice. Real-time RT-PCRs were performed using TaqMan probes specific for *Abcg5* (**A**) and *Abcg8* (**B**). Units are the relative gene expression normalized to *Gapdh*. Results are shown as means ± SEM. *p*-values were determined by Student’s *t*-test, *p < 0.05, ***p < 0.001.

In the final steps of the reverse cholesterol transport, the cholesterol half-transporters ABCG5 (ATP-binding cassette transporter G5) and ABCG8 (ATP-binding cassette transporter G8) play a critical role. Both hemi-transporters are present on the apical membrane of hepatocytes facilitating excretion of cholesterol and plant sterols into the bile and are also expressed in the intestinal epithelial cells^41,42^. Because elevated expression of both ABCG5/G8 in *Ldlr*^−/−^ mice contributes to the attenuation of diet-induced atherosclerosis^43^, we examined the expression of *Abcg5/8* in the liver of WT, *Abcc6*^−/−^, *Ldlr*^−/−^ and *Abcc6*^−/−^*;Ldlr*^−/−^ mice. We found that the deletion of the *Ldlr* gene induced increased *Abcg5/8* expression in the liver under normal dietary conditions. There was a trend towards lower expression of *Abcg5/8* in *Ldlr*^−/−^ mice lacking ABCC6 but the difference was not statistically significant with the limited number of samples available for this experiment (Fig. 7A). However, with mice fed high fat diet, the hepatic expression of *Abcg5 and Abcg8* decreased significantly (*p* = 0.020, *p* = 0.016 respectively, n = 4) in *Abcc6*^−/−^*;Ldlr*^−/−^ mice as compared to *Ldlr*^−/−^ controls (Fig. 7B), which is consistent with the reduction in plasma bile acids.

## Discussion

Several publications describing PXE patients and *Abcc6*-deficient mouse models have previously reported sporadic and circumstantial evidence of alteration of lipoprotein levels and arteriosclerosis^16,25,44–47^. However, the relationship between ABCC6, lipoproteins and calcification has never been specifically studied. This study examined this association using mouse models under normal or atherogenic conditions and compared results with human PXE patients. We found that ABCC6 deficiency caused significant changes in lipoprotein levels and enhanced atherosclerosis, which were associated with decreased cholesterol efflux from macrophages and the dysregulation of genes involved in purinergic signaling including *Nt5e*^5,6,48,49^, and in reverse cholesterol transport (ABCG5/8). The presence of pro-inflammatory conditions was likely a compounding factor. Remarkably, dyslipidemia and the development of atherosclerotic lesions occurred in a haploinsufficient manner, unlike the calcification phenotype caused by *ABCC6* mutations.

### Dyslipidemia and atherosclerosis occur independently

The possible effect of ABCC6 deficiency on lipoproteins was first described in PXE patients by Wand et al*.*^47^. The authors of this study reported an association between an *ABCC6* variant (now considered non-pathogenic^35,50,51^) and elevated HDL levels and low triglycerides. However, these results are inconsistent with what others have since described^22,31,33^. A few years later, Gorgel et al*.* obtained more solid but limited lipoprotein data using mice^25^. The results presented here have now largely expanded upon these initial observations with a more detailed analysis of both mice and humans lacking functional ABCC6. Remarkably, we observed in our mouse model with an atherogenic background (*Abcc6*^−/−^*;Ldlr*^−/−^) the formation of atherosclerotic plaque in the aorta even under normal dietary conditions and in the absence of dyslipidemia. These results suggest that plaque development occurs in these mice independently of LDL/HDL levels, and imply that other molecular, cellular and/or physiological mechanism(s) are involved. The elevated triglycerides under these normal dietary conditions were probably one of the contributing factors^52^. When these mice were challenged with high fat diets, ABCC6-specific differences in lipoprotein levels and plaque development were dramatically enhanced indicating that ABCC6 influences lipoprotein levels and atherosclerosis through independent but compounding mechanisms.

### ABCC6 influences plaque formation by modulating HDL

Although mice are the most common animal model used to study human diseases, the biology of rodents can be noticeably divergent^53^. *Abcc6*^−/−^ mice, despite the known differences with the human phenotype^54^, have been extraordinarily useful for the study of PXE^55,56^ and the development of therapeutic approaches^49,57–60^. However, mice present singular differences in lipoproteins and atherosclerosis development^61^. For this reason, the overlapping characteristics between animal and PXE patient data weighed heavily in our interpretations. Even though we could only speculate as to what the link between ABCC6 molecular function and HDL metabolism could be, we distinguished in our data and the literature several arguments suggesting that ABCC6 modulates atherosclerosis by influencing HDL quantity and/or quality^62^, as it is the most common denominator between our mouse models and human subjects.

1. We explored possible cellular mechanisms that could enhance atherosclerosis. We first examined cholesterol efflux from bone marrow-derived macrophages after lipid loading. Reduced cholesterol efflux from macrophages within the plaque is probably a significant contributing factor to the enhanced atherosclerotic plaque development we observed in the animal models and PXE patients especially in the absence of changes to LDL in humans. However, the reason behind the strictly ABCC6-dependent decrease in cholesterol efflux is unclear. Indeed, there was no significant change in *Abca1* or *Abcg1* expression and protein levels or in other related genes between control and experimental macrophages and foam cells. Furthermore, the notable absence of *Abcc6* mRNA in macrophages likely reflected an acquired phenotype that persisted *ex-vivo* after bone marrow isolation and macrophage differentiation. We and others have previously described similar situations with primary skin fibroblasts, which express little to no *ABCC6,* from PXE patients that retain altered characteristics ex-vivo^40,63,64^. An alternative explanation could be that even a minimal ABCC6 presence may exert a meaningful impact on cellular functions as Hendig and co-workers suggested^65,66^.
2. A recent study of ABCC6-deficient mice in *ApoE*^−/−^ background showed no significant changes in atherosclerosis (or arterial calcification) as compared to *ApoE*^−/−^ controls^30^. The results of this study are not necessarily discordant with ours and in fact provided some support to our overall conclusion regarding HDL. *ApoE*^−/−^ mice are a very useful and popular animal model. However, atherogenesis in mice is quite distinct from humans as HDL cholesterol is virtually absent in these mice^67^ and atherosclerosis is not a distinguishing feature of humans lacking ApoE^68^. In that sense, *Ldlr*^−/−^ mice provide a pathophysiology somewhat closer to that of humans as plaque formation is primarily driven by higher LDL and lower HDL^69^ and this is what we observed in the present study (Table 1, and Fig. 1). Since Van der Veken et al. reported no difference in atherosclerosis or lipoproteins between *Abcc6*^−/−^;*ApoE*^−/−^ mice and *ApoE*^−/−^ controls, we first suspected that ApoE could play a role in our model. Indeed, the absence of ApoE in macrophage promotes atherosclerosis without changing plasma cholesterol and we found that atherosclerosis occurred independently of dyslipidemia^70^. However, we found no significant variation in the plasma of mice or in PXE patients. Another likely possibility was that *ApoE*^−/−^ mice have very little or no circulating HDL. If ABCC6 modulates atherogenesis by its differential effect on HDL quantity (Table 1), and/or quality^62^, then ABCC6 would have less influence on plaque formation in *ApoE*^−/−^ mice. In this regard, the data from Van der Veken et al. are consistent with our results and one could regret the lack of cholesterol efflux experiment in their study.
3. Reverse cholesterol transport, which is primarily mediated by HDL, is a mechanism by which excess cholesterol is removed from peripheral tissues and delivered to the liver for either redistribution or excretion in the form of bile acids. About 95% of bile acids are reabsorbed during the digestive process. Thus, lower plasma bile acid level signals either a reduction of the enterohepatic recirculation (intestinal absorption and liver reabsorption) and/or decreased reverse cholesterol transport. Lower HDL plasma concentration, reduced cholesterol efflux from foam cells and the dramatic changes in plasma bile acids are all consistent with altered reverse cholesterol transport. The lack of change in *Sr-b1, ApoA1* and *Cyp7*α expression indicated that the canonical FXR pathway was unaffected in liver and also agrees with this interpretation.
4. As circumstantial evidence, a recent comparative study of polar and brown bear genomes identified *ABCC6* as one of 20 genes under positive selection that allowed polar bears to adapt to a lipid-rich diet and elevated plasma LDL^71^.
5. Furthermore, we found clear evidence for an ABCC6-dependent pro-inflammatory status both at the cellular levels in murine macrophages and foam cells with overproduction of CCL-2 as well at the systemic levels with elevated plasma levels of IL-6 both in PXE patients and our mouse models. The role of inflammation in atherosclerosis development is well-documented and thus these cytokines and chemokines are likely factors aggravating atherosclerosis associated with ABCC6 deficiency.

### The pluripotent physiological function of ABCC6

**Figure 8:**
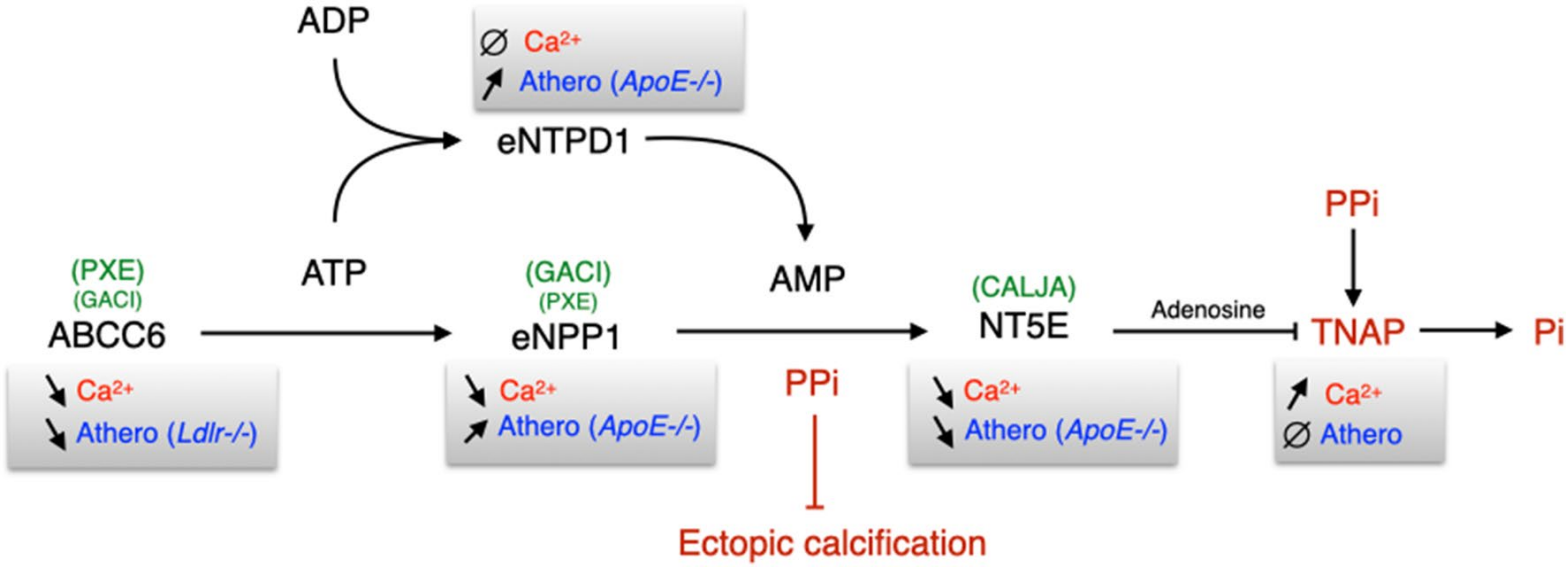
The proteins/enzymes in this pathway regulate both calcification and atherosclerosis. ABCC6 activity facilitates the cellular efflux of ATP from liver and other tissues/cells, which is quickly converted to pyrophosphate (PPi), a potent inhibitor of mineralization. Decreased plasma PPi levels cause calcification in PXE (OMIM #264800) and GACI (OMIM #614473 and #208000). NT5E activity leads to adenosine production which has numerous biological activities towards inflammation, atherosclerosis and inhibition of TNAP synthesis. TNAP degrades PPi into inorganic phosphate (Pi), an activator of calcification which leads to vascular calcification in Calcification of Joints and Arteries patients (CALJA, OMIM #211800). In addition to ABCC6, both ENPP1 and NT5E functions have been linked to atherosclerosis development in *ApoE*^−/−^ mice. ENTPD1 function overlaps with that of ENPP1 and also plays a role in atherosclerosis in mice lacking ApoE, though this ectoenzyme does not seem to regulate ectopic calcification. Symbols: ∅ : no known effect; ➚: increase; ➘: decrease.

The emerging model that ABCC6 is a modulator of the dynamic equilibrium of extracellular nucleotides (ATP) and adenosine^5,6,9,48,49,72^ is an indication that ABCC6 physiological function extends beyond the regulation of mineralization. Indeed, ABCC6 activity facilitates the cellular efflux of ATP from liver and other tissues/cells^11,12^, which is sequentially converted by ENPP1 and NT5E (*i.e.CD73*) into pyrophosphate (PPi) and adenosine, two potent inhibitors of mineralization^5,7^. However, ATP and adenosine have numerous other biological activities, notably towards inflammation^73–75^ and atherosclerosis^76–80^. Remarkably, the other enzymes downstream of ABCC6 (Fig. 8) that regulate ectopic calcification in PXE, GACI and CALJA^4,5,7,9,13,48,49,81^ have also been shown to play a role in atherosclerosis development^76–79^. Of note, ENTPD1 (*i.e.* CD39)*,* which has a molecular function similar to that of *ENPP1,* influences atherosclerosis^82,83^, though this ectoenzyme does not regulate calcification (G. Kauffenstein, personal communication). Our current data on ABCC6 fit this pattern of dual function in this pathway.

In conclusion, the present study reveals a novel physiological role for ABCC6, influencing plasma lipoproteins and atherosclerosis in a haploinsufficient manner, with significant penetrance.

## Supplementary Information

Supplementary Information.
