# Supplementary material for: ABCC6 deficiency promotes dyslipidemia and atherosclerosis
Source: Sci Rep. 2021 Feb 16;11:3881. doi: 10.1038/s41598-021-82966-y (PMC7887252; doi:10.1038/s41598-021-82966-y)
Supplement: Supplementary file 1 — Supplementary Information. [file 41598_2021_82966_MOESM1_ESM.pdf]

ABCC6 deficiency promotes dyslipidemia and atherosclerosis

Christopher Brampton[1,2], Viola Pomozi[1], Li-Hsieh Chen[1], Ailea Apana[1], Sara McCurdy[3, 4], Janna Zoll[1], William A. Boisvert[3], Gilles Lambert[5], Daniel Henrion[6], Simon Blanchard[7,8], Sheree Kuo[9], Georges Leftheriotis[10,11], Ludovic Martin[12,13] and Olivier Le Saux[1*]

SUPPLEMENTAL FIGURE and LEGENDS

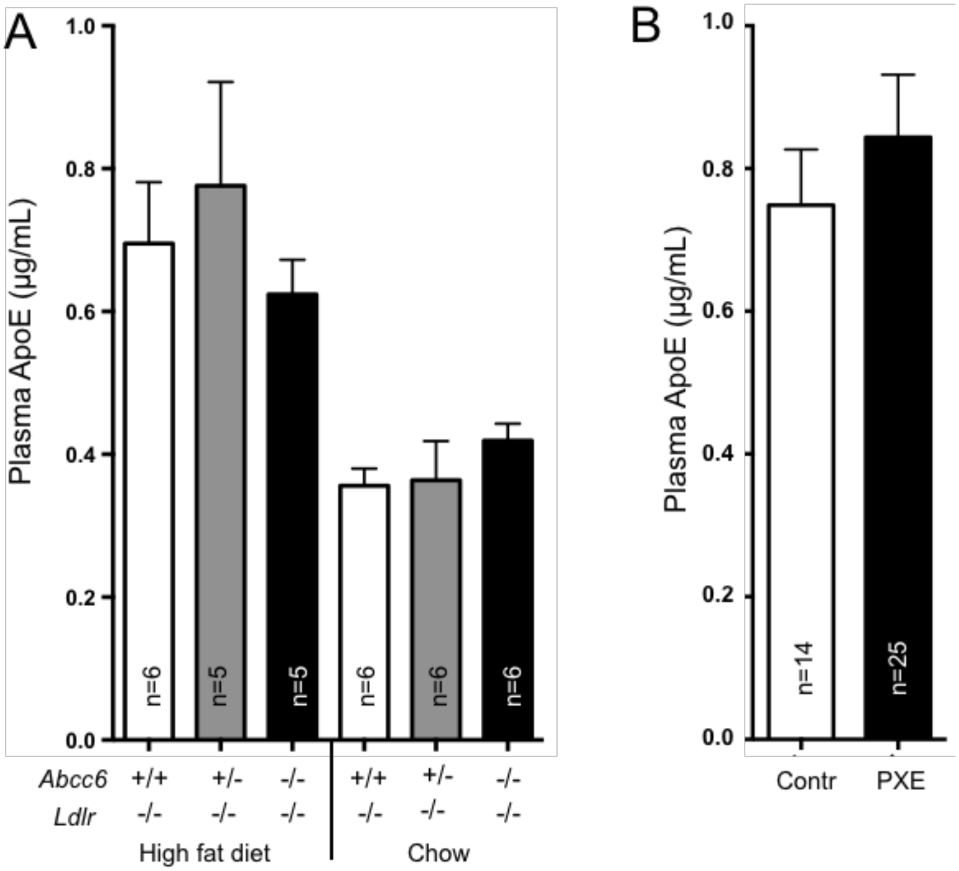

Figure 1: ELISA quantification of plasma ApoE in mice and human samples. The fasting levels of plasma ApoE were quantified with a commercially available kit. The number of individual samples are indicated. Results are shown as means +/- SEM.

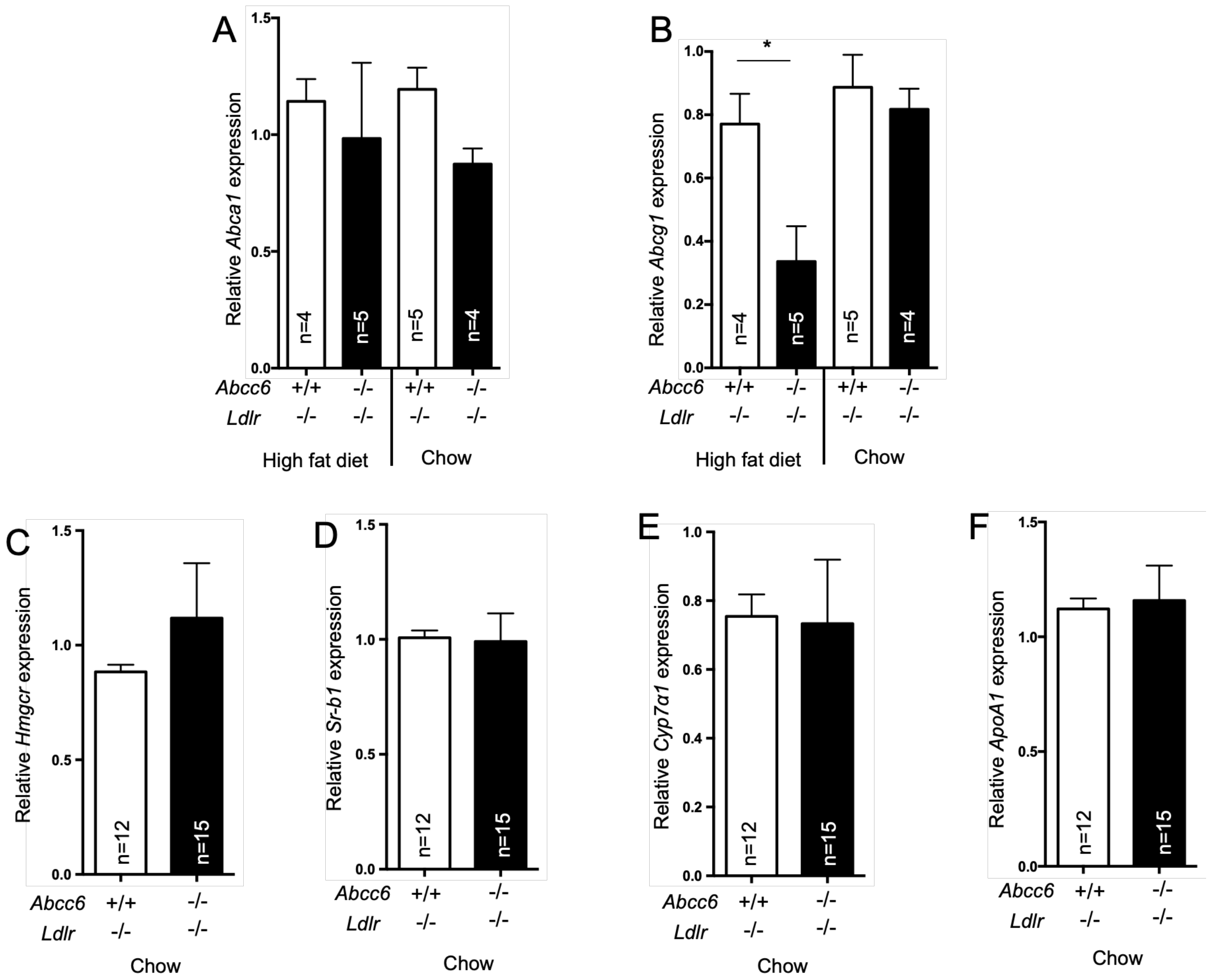

Figure 2: Expression of several genes related to cholesterol and bile acid metabolisms in the liver of *Ldlr*$^{-/-}$ mice lacking *Abcc6* fed normal or high fat diets. Real-time RT-PCRs were performed using TaqMan probes specific for *Abca1* (**A**), *Abcg1* (**B**), *Hmgcr* (**C**), *Sr-b1* (**D**), *Cyp7*α*1* (**E**) and *ApoA1* (**F**) cDNAs and the *Gapdh* housekeeping gene for normalization. Units are a ratio of the relative gene expression to *Gapdh*. *Abcg1* expression was found to be significantly downregulated in *Abcc6*$^{-/-}$;*Ldlr*$^{-/-}$ mice fed high fat diet. The numbers of mice used for individual experiments are shown. Results are shown as means ±SEM. *p*-values were determined by Student's *t*-test, * p<0.05.

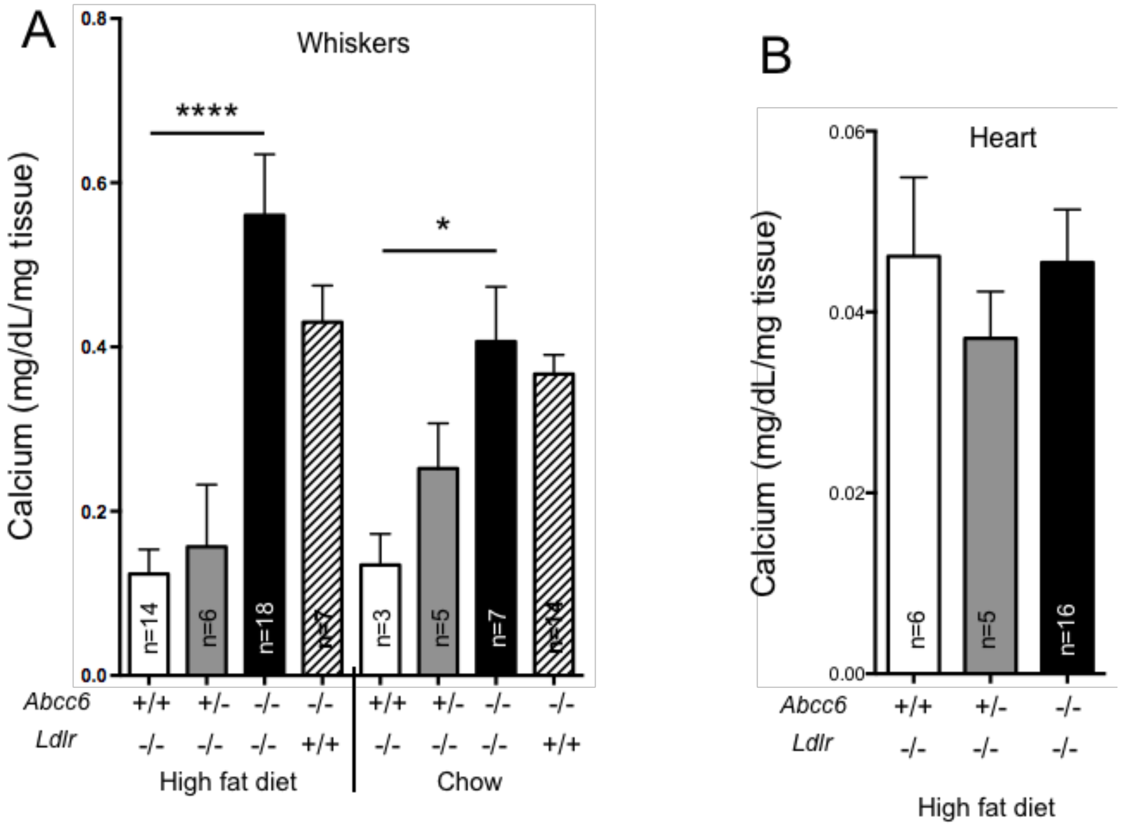

Figure 3: Whisker and cardiac calcification in *Ldlr*$^{-/-}$ mice lacking *Abcc6*. The level of calcification in the whiskers was measured as total calcium content normalized to the weight of the tissue. Littermate mice were maintained on an atherogenic diet (high fat) or regular chow for 16 weeks. As shown the level of vibrissae (whiskers) calcification induced by *Abcc6*-deficiency was significantly elevated only in mice lacking both *Abcc6* alleles (as expected) but was otherwise not influenced by the diet. There was also no difference in cardiac calcification in mice fed high fat diet. Mouse numbers are indicated. Results are shown as means ±SEM. * p<0.05, ** p<0.01, *** p<0.001.

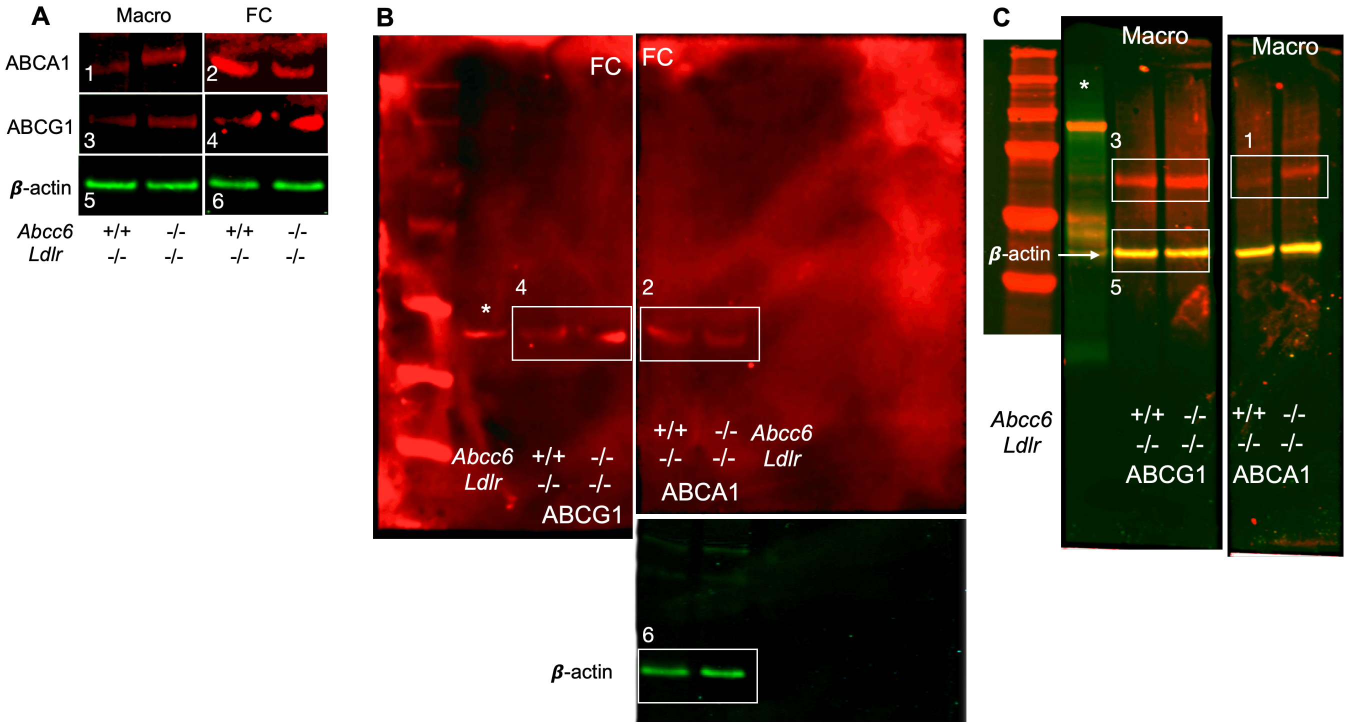

Figure 4: Full-length sections of western blot images from Figure 4C. **A:** A reproduction of Fig. 4C with representative western blot images showing the levels of both ABCA1 and ABCG1 expression (red signal) in foam cells (FC) as compared to macrophages (Macro). β-actin (green) was used as loading control. **B and C:** Sections of the full-length images. The bottom images of **C** are overexposed to reveal the lesser exposed upper red bands *vs* the green beta-actin controls. The Asterix on panel **B** and **C** correspond to lanes loaded with unrelated samples. Each cropped section of the blots is marked by numbers and referenced on the full-length images.

Supplemental Table

Table 1: Lipoprotein profile analysis of mouse models and human subjects lacking ABCC6.

**Diets**	**Genotype**		**LDL**		**HDL**		
	Ldlr	***Abcc6***	**Small**	**Large**	**Small**	**Intermediate**	**Large**
			mg/dL	mg/dL	mg/dL	mg/dL	mg/dL
**High fat**	**-/-**	**+/+**	5.6 ±2.1	96.3 ±20.2	7.4 ±6.2	62.8 ±6.3	59.0 ±7.5
**High fat**	**-/-**	**+/-**	**24.1 ±4.7 ***	108.8 ±12.6	1.5 ± 1.5	36.3 ±10.9	50.0 ±12.6
**High fat**	**-/-**	**-/-**	**33.6 ±6.4 *****	126.3±12.3	**0**	50.3 ±13.4	75.8 ±7.1
**Chow**	**-/-**	**+/+**	11.5 ±1.6	18.1 ±2.8	0.6 ±0.4	38.8 ±4.5	20.6 ±1.9
**Chow**	**-/-**	**+/-**	12.9 ±1.1	21.5 ±1.6	0.6 ± 0.6	61.8 ±14.7	30.0 ±7.1
**Chow**	**-/-**	**-/-**	11.0 ±1.0	20.7 ±2.0	0.7 ±0.7	40.7 ±10.9	24.0 ±9.0
**High fat**	**+/+**	**-/-**	1.2 ±0.2	4.5 ±0.3	5.0 ±2.5	68.4 ±11.5	22.2 ±4.9
**High fat**	**+/+**	**+/+**	0.5 ±0.3	3.9 ± 0.8	3.4 ±0.6	103.6 ±16.4	36.0 ± 4.4
**Chow**	**+/+**	**-/-**	**1.7 ±0.3** #	**4.3 ±0.3** ##	1.4 ±0.9	**54.4 ±3.8** #	**16.6 ±3.5**#
**Chow**	**+/+**	**+/+**	0.5 ±0.2	2.0 ±0.7	1.3 ± 0.9	71.0 ± 4.4	29.5 ±4.2

	**LDL**		**HDL**		
	**Small**	**Large**	**Small**	**Intermediate**	**Large**
	mg/dL	mg/dL	mg/dL	mg/dL	mg/dL
**Healthy Controls**	0.18 ±0.11	28.2 ±-3.05	12.4± 1.12	29.5 ±2.25	26.6 ±3.21
**PXE Patients**	0.17 ±0.08	26.7 ±3.32	**8.2 ± 0.61** ##	**24.3 ±0.99** #	**18.3 ±1.49** ##

Bold shows significant differences with controls.
Mice age: 5 months-old, n=3 to 8.
p-values were determined by one-way ANOVA and Tukey's post hoc test (*) or Student's *t*-test (#).
*, # p<0.05, **, ## p<0.01, ***p<0.001 **** p<0.0001.
